# CARD domain of rat RIP2 kinase: Refolding, solution structure, pH-dependent behavior and protein-protein interactions

**DOI:** 10.1371/journal.pone.0206244

**Published:** 2018-10-23

**Authors:** Sergey A. Goncharuk, Lilya E. Artemieva, Valentin M. Tabakmakher, Alexander S. Arseniev, Konstantin S. Mineev

**Affiliations:** 1 Shemyakin–Ovchinnikov Institute of Bioorganic Chemistry, Russian Academy of Sciences RAS, Moscow, Russian Federation; 2 Moscow Institute of Physics and Technology, Institutsky per., Dolgoprudnyi, Russian Federation; George Washington University, UNITED STATES

## Abstract

RIP2, one of the RIP kinases, interacts with p75 neurotrophin receptor, regulating the neuron survival, and with NOD1 and NOD2 proteins, causing the innate immune response against gram-negative and gram-positive bacteria via its caspase recruitment domain (CARD). This makes RIP2 a prospective target for novel therapies, aimed to modulate the inflammatory diseases and neurogenesis/neurodegeneration. Several studies report the problems with the stability of human RIP2 CARD and its production in bacterial hosts, which is a prerequisite for the structural investigation with solution NMR spectroscopy. In the present work, we report the high yield production and refolding protocols and resolve the structure of rat RIP2 CARD. The structure reveals the important differences to the previously published conformation of the homologous human protein. Using solution NMR, we characterized the intramolecular mobility and pH-dependent behavior of RIP2 CARD, and found the propensity of the protein to form high-order oligomers at physiological pH while being monomeric under acidic conditions. The oligomerization of protein may be explained, based on the electrostatic properties of its surface. Analysis of the structure and sequences of homologous proteins reveals the residues which are significant for the unusual fold of RIP2 CARD domains from different species. The high-throughput protein production/refolding protocols and proposed explanation for the protein oligomerization, provide an opportunity to design the stabilized variants of RIP2 CARD, which could be used to study the structural details of RIP2/NOD1/NOD2 interaction and perform the rational drug design.

## Introduction

RIP kinase family comprises the proteins that take part in death receptor signaling and innate immunity. RIP kinases bind various TNFR family members and NOD receptors, activating the NFκB and MAP signaling cascades [1,2]. RIP2 (RICK, RIPK2), one of the RIP kinases, interacts with p75 neurotrophin receptor [3], regulating the neuron survival, and with NOD1 and NOD2 proteins, causing the innate immune response against gram-negative and gram-positive bacteria [4]. This makes RIP2 a prospective target for novel therapies, aimed to modulate the inflammatory diseases and neurogenesis/neurodegeneration [5].

RIP2 has a typical two-domain structure, the N-terminal part of the protein possesses the catalytic kinase activity, while the C-terminal part is a caspase recruitment domain (CARD), belonging to the death domain superfamily [6]. Kinase activity is not required for the NFκB signaling but is necessary to stabilize the protein and to activate the MAP kinase pathway [7,8]. CARDs, in turn, are essential for the interactions of RIP2 with its partner receptors, both NODs and p75 [3,9,10]. Two domains are linked together by a presumably unstructured segment of ~140 amino acids, a so-called intermediate domain. The full-size RIP2 protein is in the monomer-dimer equilibrium in solution [11].

The structural data regarding the RIP2 until recently was very limited. The first structure of the kinase domain [12] and NMR structures of human RIP2 CARD in the apo state and in complex with the death domain of p75NTR were published in 2015 [13]. Interfaces of protein-protein interactions, accomplished by RIP2 CARD were also probed by mutagenesis for NOD1 [14], NOD2 [15] and p75NTR [16], however, no structure of NOD1 or NOD2 complex with RIP2 CARD was obtained. Several studies report the problems with the stability of human RIP2 CARD and its production in bacterial hosts, which is a prerequisite for the structural investigation with solution NMR spectroscopy [13,15]. Fridh et al. [15] were unable to produce the soluble mouse RIP2 CARD domain for the structural study and had to co-express it with the NOD2 domain for the pull-down assay. Despite the fact that Lin et al. [13] succeeded in the bacterial production of human RIP2 CARD and even resolved its structure, authors faced the solubility problems; the production protocol is described briefly and not clearly, and therefore cannot be reproduced. In the present work, we focus on the CARD of rat RIP2 kinase, highly homologous to the human protein. Our aim is to first establish the protocol of protein production and then to thoroughly characterize its structure and stability, to obtain an object, applicable for the studies of functionally important protein-protein interactions.

## Materials and methods

### Protein expression and purification

Four different expression constructs for RIP2 CARD domain (RIP2CARD) were tested. Several variants of pGEMEX-1 vector with the desired N-terminal tag (His-tag, Thioredoxin, SUMO, and GST) cloned into the NdeI/BamHI restriction sites were engineered. To remove the N-terminal tag the thrombin cleavage site was incorporated into all constructs. The RIP2CARD gene was assembled using chemically synthesized oligonucleotides, digested with BamHI and HindIII endonucleases and ligated into all vector variants.

The expression vectors were transformed into E. coli BL21(DE3)pLysS strain and were streaked onto the LB agar plates. For each variant, several colonies (about 1 colony per 10 ml of medium) were picked from the plates next evening, inoculated into M9 media in the presence of ampicillin (100 μg/mL) and cultured at 28°C and 250 rpm overnight until the culture reached an OD600 of ~0.6. Protein expression was induced by IPTG. To find the best cultivation conditions three different IPTG concentrations (0, 0.1 and 1 mM) and two temperatures (37°C and 13°C) were tested. The cells were incubated overnight, harvested by centrifugation at 5000 g for 7 minutes and stored at -20°C. To estimate the protein solubility the cell pellet was resuspended in buffer (20 mM Tris-HCl, pH 8.0, 100 мМ NaCl, 100 μM PMSF, 1 mM EDTA, 0.5% Triton X-100) and lysed by ultrasonication on ice. The cell debris and non-soluble proteins were removed by centrifugation at 14000 g for 20 minutes.

To prepare the protein for the refolding procedure the His-tagged variant was used. Protein expression was induced by 0.1 mM of IPTG and cultivation was continued at 37°C. The cells were harvested by centrifugation at 5000 g for 7 minutes and stored at -80°C. The cell pellet was resuspended in lysis buffer (50 mM Tris-HCl, pH 8.0, 500 мМ NaCl, 10 mM β-mercaptoethanol (β-Me), 100 μM PMSF), lysed by ultrasonication on the ice and centrifuged at 14000 g for 1 hour. The pellet was resuspended in buffer containing 50 mM Tris-HCl, pH 8.0, 500 мМ NaCl, 10 mM β-Me, 10 mM imidazole and 8 M urea, incubated for 1 hour and the cell debris and non-soluble proteins were removed by centrifugation at 14000 g for 1 hour followed by filtration through the membrane with 0.22 μm pores. The soluble fraction was bound to Ni-sepharose HP resin (GE, USA), washed with the same buffer containing 30 mM imidazole and eluted with the buffer containing 500 mM imidazole. All buffers used during the purification contained 10 mM β-Me.

### Refolding

The concentration of protein after IMAC was usually around 2 mg/ml, otherwise, it was concentrated to that value. All dialysis procedures were carried out in the dialysis tubes with 14 kDa weight cut-off. The buffer volume was taken in the 400-fold excess over the sample volume. The duration was 18–24 h at 4°C or RT with vigorous stirring by the magnetic mixer. The straightway dialysis at pH 7.5–8.5 was conducted using buffer A containing: 50 mM Tris (adjusted to the desired pH with HCl), 50 mM NaCl, 1 mM NaN3 and 5 mM β-mercaptoethanol (β-Me). For the dialysis at pH 6 the buffer containing 20 mM NaPi, 50 mM NaCl, 1 mM NaN3 and 5mM β-Me was used. For the dialysis procedures at pH 4.5–5 buffer containing 10 mM NaOAc, 1 mM NaN3 and 0.5 mM tris(2-carboxyethyl)phosphine (TCEP) was used. Stepwise dialysis was into the buffers containing reducing concentration of urea (8M -> 4M -> 2M -> no urea) at pH 8 and pH 5.

To assess the impact of slight dilution prior to dialysis, the protein sample was diluted 11-fold with buffer A at pH 7.5 and incubated overnight. To investigate the effect of arginine, the protein sample was diluted 6-fold with buffer A with 0.5 M arginine and incubated overnight. Then, the standard straightway dialysis was carried out.

In dilution experiments the protein was added to the buffer (10 mM NaOAc, 1 mM NaN3, 0.5 mM TCEP with or without 50 mM NaCl) dropwise at 1 ml/h with the peristaltic pump at 4°C and pH 4–4.5.

The final refolding was performed at 4°C by 130-fold dilution in the buffer containing 10 mM NaOAc, pH 4.2, 0.5 mM TCEP and 1 mM NaN3. After the gentle stirring at 4°C overnight, the protein sample was concentrated to ~10 mg/ml, using the 10 kDa MWCO Amicon ultrafiltration discs (pressure-based sample concentration). The quality of refolding was controlled using CD and NMR spectra.

### NMR spectroscopy

Unless otherwise specified, all NMR spectra were recorded on 300 μM RIP2CARD samples, at pH 4.2, 1 mM TCEP and 30°C on Avance III 800 MHz spectrometer (Bruker Biospin, Germany), equipped with triple resonance cryogenic probe. Chemical shift assignment was made manually using the following experiments: HNCO, HN(CA)CO, HNCA, HN(CO)CA, HNCACB, CBCA(CO)NH, H(CCCO)NH, (H)CC(CO)NH, H(C)CH-TOCSY, 4D HCCH-TOCSY, 3D-1H,15N-NOESY-HSQC. Aromatic sidechains were assigned using the (H)CCH-COSY and (Hb)Cb(CgCC)H spectra [17,18]. When possible, BEST-TROSY versions of the experiments were utilized with 0.45 s recycling delay [19]. All spectra were recorded with non-uniform sampling in the indirect dimensions and processed using the compressed sensing approach in qMDD software [20]. ^3^J_HNHA_ were measured from the J-quantitative HNHA experiment [21], ^3^J_CCo_ and ^3^J_NCo_ couplings were measured from the spin-echo difference constant-time HSQC spectra [22,23]. Structure of the protein was calculated based on the distance restraints derived from the 3D 1H,15N-NOESY-HSQC and 1H,13C-NOESY-HSQC spectra acquired with 80 ms mixing time and dihedral restraints (𝝋, 𝜒_1_) obtained from the analysis of J-couplings. The calculation was performed in CYANA 3.97 software [24] using the automated NOE assignment followed by the manual assignment of the remaining cross-peaks. To study the backbone mobility the cross-correlated relaxation rates of amide groups were measured, as reported [25] and converted to the correlation times of rotational diffusion. Structures were analyzed in the MOLMOL software [26] and the PyMOL Molecular Graphics System, Version 2.0 Schrödinger, LLC. Obtained chemical shifts and 20 best structures were deposited to BMRB and PDB databases under the accession codes 34294 and 6GWM, respectively. To determine the pKa of ionogenic groups we followed the chemical shifts of Asp Cβ, Glu Cγ and Cα of the C-terminal residue, varying pH in the range 3.3–5.1 in the absence of salt. The obtained dependence was then approximated with the most simple model: *δ_c_* = (*δ_a_* + *δ_b_*10^(*pKa−pH*)^)/(1 + *δ_b_*10^(*pKa−pH*)^), where *δ_c_* is the current chemical shift, and *δ_a_* and *δ_b_* are chemical shifts of the nuclei in the protonated and the deprotonated states, respectively. Analysis of the protein electrostatic properties was performed with the Protein Surface Topography approach [27,28], implemented in the in-house software IMPULSE. NOD1 and RIP2 CARD domains were spatially aligned, the 3D distribution of electrostatic potential on the molecular surface of NOD1 CARD and RIP2 CARD was computed using the AMBER99SB-ILDN parameters set [29], and then transformed to the 2D spherical projection maps.

## Results

### Protein expression

Protein production in the soluble form is certainly the most preferred strategy since the development of an effective refolding procedure often takes a lot of time and resources, but the success is not guaranteed. Therefore, several expression constructs were first screened to produce RIP2CARD. We tested the following N-terminal tags: Thioredoxin, SUMO, GST and His-tag. Additionally, we studied the effect of the His-tag position at either N- or C-terminus of RIP2CARD, but no significant changes were observed. To assess the solubility, we used the non-denaturing detergent, Triton X-100. It facilitates the cell lysis and allows the clearer interpretation of the protein accumulation pathway (as inclusion bodies or not). Thus, we exclude the small aggregates of RIP2CARD or co-precipitation due to the hydrophobic interactions, e.g. with the membrane (membrane-associated proteins).

To compare the expression levels and protein solubility for all hybrid constructs and cultivation conditions, the SDS-PAGE analysis was performed. First of all, the overall protein yields were compared (S1 Fig, Table 1). A high-level expression for all constructs and cultivation conditions was observed. It is noteworthy that the expression levels for thioredoxin- and His-tagged constructs cultivated at 13°C and 37°C were approximately the same, while for SUMO and especially for GST constructs significantly lower yields were achieved, growing cells at 13°C. Despite the fact that the absolute yield of His-tagged construct was smaller, the target protein (RIP2CARD) represents the major part of this hybrid, while in all other fusions the RIP2CARD represents less than one half. Thus, in terms of the target protein yields, we can conclude that Thioredoxin- and His-tagged hybrids are the best constructs for cultivation at any temperature.

**Table 1:** Normalized approximate yields of RIP2CARD per 1 L of M9 medium for different constructs and cultivation temperatures.

Fusion construct	total (soluble), mg/L at 37°C	total (soluble), mg/L at 13°C
Trx-RIP2CARD	40^a^ (0)	40 (15)
SUMO-RIP2CARD	40 (0)	15 (10)
GST-RIP2CARD	20 (0)	3 (1)
H6t-RIP2CARD	30 (0)	30 (1)

^a^The yields were estimated from the SDS-PAGE gels (see S1 Fig).

Apart from the overall protein quantity, the amount of soluble fraction is also essentially important. It turned out that the major part of the target protein is insoluble at 37°C for all constructs, while the accumulation of soluble form is observed at low temperature (13°C). The most considerable changes were found for thioredoxin and SUMO constructs. About one third and a half of the target protein, respectively, was soluble, while no significant amount of soluble His-tagged construct was produced. The temperature dependence could be explained by the hydrophobic interactions and presence of the fusion partner. At low temperature, the hydrophobic interactions become weaker which allows the protein to form the correct intramolecular contacts and adopt the native fold. The fusion partner, like thioredoxin, can conceal the hydrophobic areas on the RIP2CARD surface, which does not allow the oligomers formation.

Thus, we showed that a soluble form of RIP2CARD can be obtained with the fusion partner, but no more than 15 mg of the target protein (RIP2CARD) per liter of medium, while the total yield can reach 30–40 mg per liter. In addition, even these reduced amounts of soluble chimeric proteins were unstable and precipitated after the first purification step by IMAC. In this regard, we decided to develop a protein refolding procedure from the inclusion bodies.

### Refolding protocol for RIP2CARD from the inclusion bodies

The refolding procedure appears to be one of the major bottlenecks in getting the natively structured and active recombinant protein. In the case of RIP2CARD, the refolding is aimed to eliminate the strong solvents from the previous step of production protocol. To refold the purified RIP2CARD, we employed two most widely used and efficient approaches: dialysis and dilution. Theoretical isoelectric point of RIP2CARD protein is 8.8, therefore we carried out the refolding process at pH below the pI value. We tested several protocols for the specified pH range. Our first attempts included the straightway dialysis at “native” pH (7.5–8.5). Under these conditions, we observed the intense protein aggregation. The concentration of RIP2CARD after dialysis was below 0.5 mg/ml and could not be increased by any kind of the concentrating procedure. To make the refolding even more gradual, we tried the stepwise dialysis approach, reducing the amount of urea in the dialysis buffer every 12–18 hours (6 M, 4 M, 2 M, and without urea). At last two steps of the buffer switch (2 M and without urea), the protein aggregation was detected. Since the protein folding may be affected by the protein-protein interactions, induced by the high concentration, we applied several approaches to avoid the aggregation: addition of arginine and slight dilution prior to the dialysis. Arginine is known to suppress the protein aggregation and improve the refolding yields [30]. Unfortunately, all listed techniques did not result in the yield of the folded protein in sufficient amounts. RIP2CARD precipitated and its concentration in solution could not be raised above 0.5 mg/ml. Additionally, we tried to dialyze the sample at two different temperatures (4°С and RT) but no significant changes were observed.

Thus, we assumed that the refolding of RIP2CARD at neutral pH is impossible, therefore, the refolding under acidic conditions was investigated. The protein contains the polyhistidine tag and several acidic residues, therefore, the pH reduction allows to change substantially the overall charge of the molecule and prevent the precipitation due to the enhanced electrostatic repulsion. With this respect, we performed first several dialysis attempts at pH 6.0 (below the pKa of His), 5.0 (close to the pKa of Glu) and 4.5 (close to the pKa of Asp). All our attempts to refold RIP2CARD at pH 5 and 6 did not succeed, however, with the dialysis at pH 4.5, we achieved the RIP2CARD concentration, sufficient for the NMR study. CD spectroscopy confirmed the native secondary structure of the protein. After the dialysis process, the sample retained 2 mg/ml of RIP2CARD, however, concentrating resulted in the protein aggregation above 4 mg/ml. The much better results were obtained using the dropwise dilution at pH 4.5 and 4.0, the latter conditions allowed to achieve the concentration of folded RIP2CARD as high as 10 mg/ml. Thus, our data clearly shows that the most important parameter for RIP2CARD folding is pH and, finally, we found that the best refolding conditions are dilution (down to 15 μg/ml) in a salt-free buffer (10 mM NaOAc, pH 4.2, 0.5 mM TCEP and 1 mM NaN3). All refolding attempts are summarized in the Table 2. With all aforesaid, we can conclude that the most simple and efficient way to produce the RIP2CARD is its expression as inclusion bodies followed by the simple refolding.

**Table 2:** Results of the attempts to refold the RIP2CARD from *E*. *coli* inclusion bodies using various protocols.

Approach	pH	Additional details	Concentration^a^, mg/ml
dilution	4	-	9
	4.5	-	6
dialysis	4.5	-	4
	5	-	< 1
		Stepwise dialysis 6 M, 4 M, 2 M and without urea	< 1
	6	-	< 1
	7.5	-	< 0.5
		x11 dilution before dialysis	< 0.5
		x6 dilution with 0.5 M arginine before dialysis	< 0.5
	8	Stepwise dialysis 6 M, 4 M, 2 M and without urea	< 0.5
	8.5	-	< 0.5

^a^The maximal concentration of soluble protein, which can be achieved after the refolding process

### Structure and mobility of RIP2CARD

**Fig 1:**
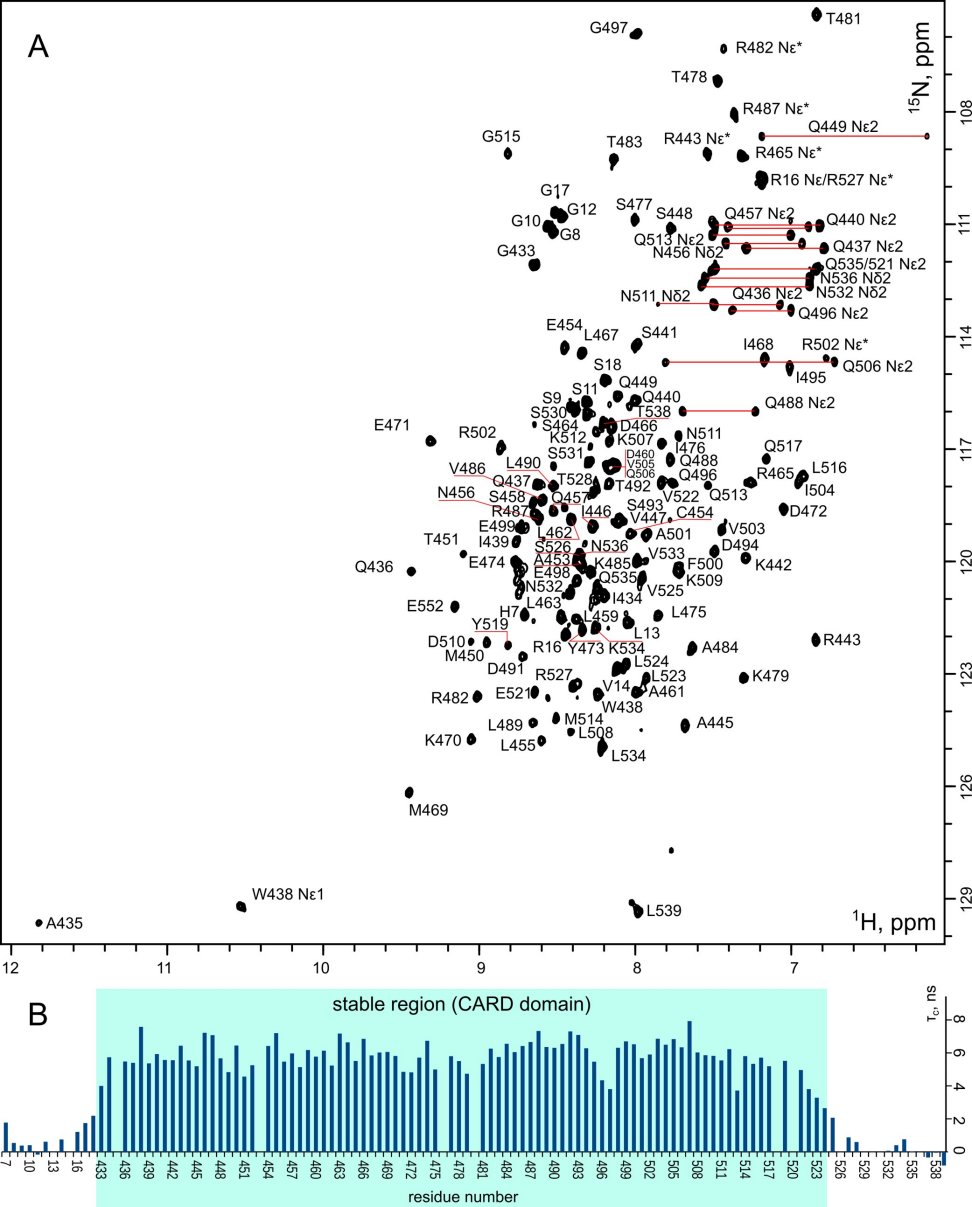
**A** - ^1^H,^15^N-HSQC spectrum of 300 μM RIP2CARD acquired at pH 4.5, 30°C. Sidechain NH_2_ groups of Asn and Gln are indicated by thick red lines and folded peaks from the Hε/Nε groups of Arg are marked by asterisks. The assignment is shown on the spectrum, peaks from the polyhistidine tag (MHHHHHHGSGSGLVPRGS) have numbers 1–18. **B**—The effective rotational correlation times of RIP2CARD N-H vectors, measured based on the cross-correlated relaxation rates.

The sample of refolded ^13^C/^15^N-labeled RIP2CARD at pH 4.2 was used to solve the structure of the protein in solution. The quality of NMR spectra was excellent (Fig 1B), which allowed obtaining 95% of possible chemical shift assignments. Only some residues of the N-terminal polyhistidine tag were not assigned. The input data for the structure calculation included 3064 NOE cross-peaks and 123 J-couplings, converted to the dihedral angle restraints. Using the automated NOE assignment, as implemented in CYANA package, the NOE peaks were converted to 1807 distance restraints. According to the analysis of the NMR relaxation parameters (Fig 1B), protein tumbles with the correlation time of 5.9 ns, which corresponds to the monomeric state of the domain, and the stable part of RIP2CARD includes 91 residues, G433-L524. Therefore, we removed the restraints for the flexible N- and C-terminal regions of the protein and finished with 1678 distance (301 long-range) and 126 dihedral angle restraints, which corresponds to 19.8 restraints per residue and should provide the overall high quality of the NMR structure.

**Fig 2:**
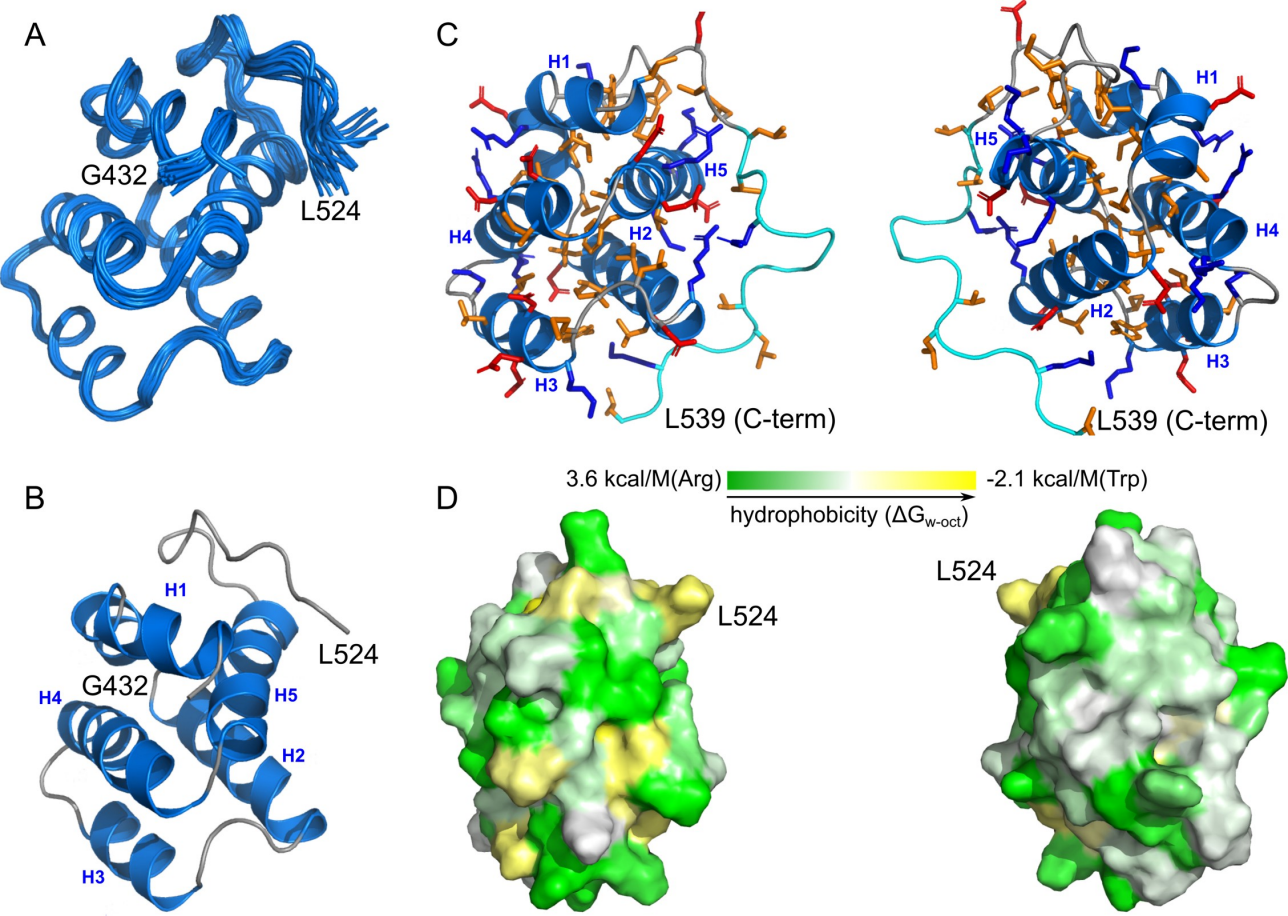
Structure of RIP2CARD. **A**—20 best structures of the stable core of RIP2CARD (432–524) are superimposed over the backbone atoms. **B**—the structure of RIP2CARD in the ribbon representation. **C—**180-degree stereoview on the structure of RIP2CARD, shown with the unstructured C-terminal tail (cyan, 525–539). Heavy atoms of some sidechains are shown by red (negatively charged), blue (positively charged) and orange (hydrophobic) sticks. **D**—the contact surface of the stable core of RIP2CARD is painted according to its hydrophobicity. Green color corresponds to the polar sidechains, while yellow stands for the hydrophobic. White and Whimley hydrophobicity scale is used [32].

The resulting structure demonstrates an almost typical fold of a protein from the death domain family (Fig 2). Protein chain forms five α-helices: kinked first helix I434-S441 (H1a) and R443-Q449 (H1b), E452-R465 (H2), K470-S477 (H3), R482-Q496 (H4), and E498-N511 (H5). Unlike many other CARD or death domains [31] that contain an additional sixth helix, the last 13 residues of the RIP2CARD do not reveal the presence of any elements of regular secondary structure except for the two successive type I β-turns (G513-L516, M514-Q517), but the region is stable, according to the 15N relaxation analysis. The Q517-P518 peptide bond is in the cis configuration, as revealed by the chemical shift analysis and strong NOE contact between the corresponding Hα atoms. The structure of the C-terminal part of the domain is unusual and may be considered as a unique feature of the RIP2 CARD since the similar fold is observed only for the highly homologous human RIP2 protein [13]. The conformation is stabilized by 52 backbone-backbone and at least two sidechain-backbone hydrogen bonds. The structure is well defined and is characterized by the backbone RMSD of 0.43 A for the region with the regular structure (I434-Q517) and 0.73 A for the full domain. Full statistics of the input data and obtained structure are provided in S1 Table. The detailed analysis of the obtained structure reveals that the core of the protein is highly hydrophobic, while the surface is mostly hydrophilic and contains many charged residues that may engage in several salt bridges. Hydrophobic chains are exposed on one face of the protein and are located mainly in the unstructured C-terminal region.

### pH-dependent behavior of RIP2CARD

As we have already mentioned, RIP2CARD can be refolded only at acidic pH, and the structure is determined at pH 4.2, which is far from the physiological values. On the other hand, the protein is rich with charged sidechains, our construct contains 16 positively charged and 15 negatively charged residues (including the N- and C-termini), as well as 6 histidines in the expression tag, which makes pH an important factor that may influence the behavior of the protein. In addition, all functionally important interactions, accomplished by the domain need to be studied at neutral pH. For that reason, we performed the pH titration experiments, to establish the relevance of the determined structure and to obtain the conditions, close to physiological.

**Fig 3:**
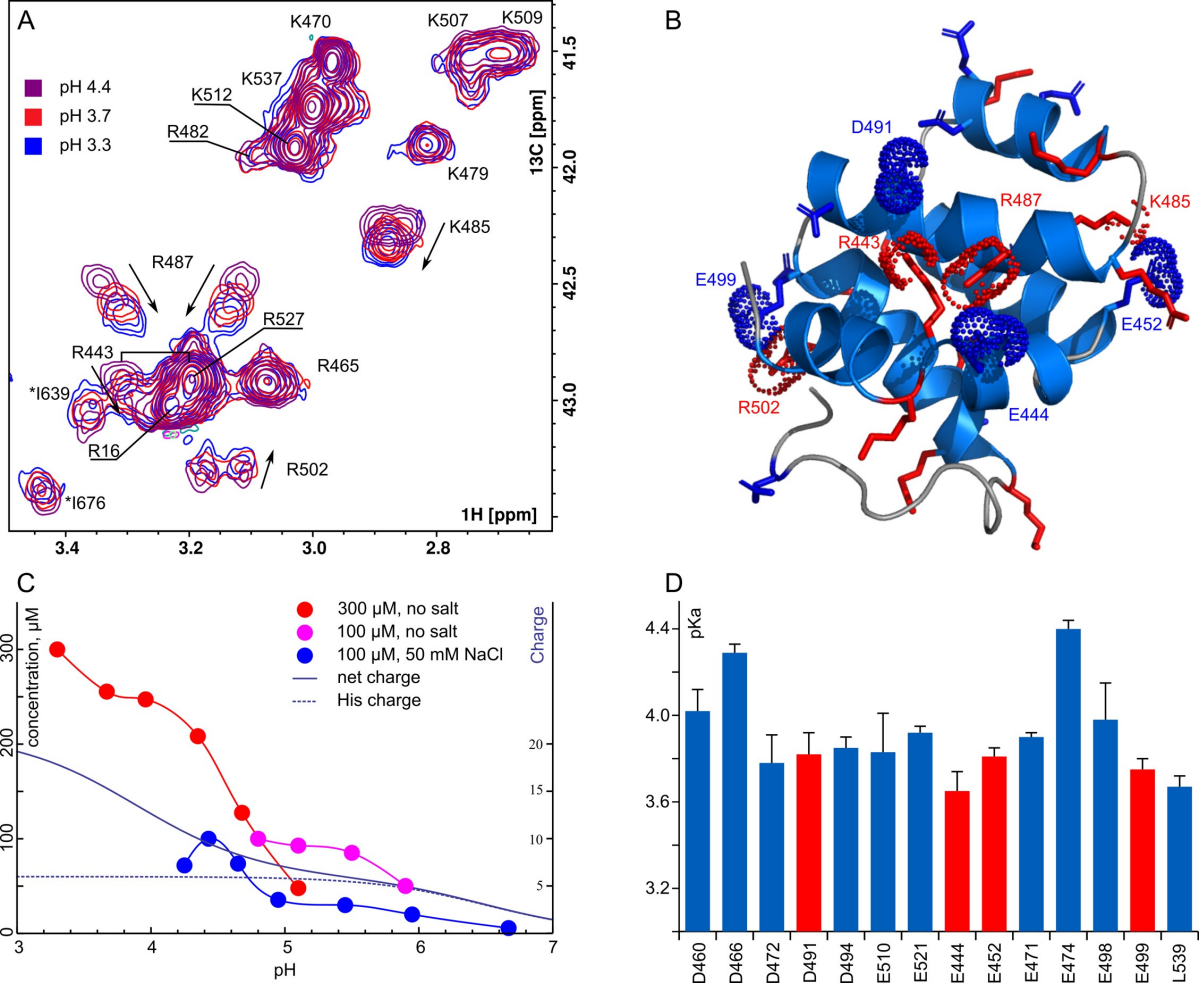
pH-dependent behavior of RIP2CARD. **A—**Overlay of the region of 13C-CT-HSQC spectra of RIP2CARD, containing the cross-peaks from the Arg and Lys sidechains, recorded at pH 3.3 (blue), 3.7 (red) and 4.4 (magenta). Assignment of signals is indicated, asterisks denote the folded cross-peaks from the CαH moieties. **B—**structure of RIP2CARD with highlighted charged sidechains, taking part in the salt bridges.**C**—concentration of monomeric RIP2CARD (the relative intensity of W438 sidechain cross-peak in HSQC) is plotted as a function of ambient pH for 300 μM sample without salt (blue circles), 100 μM sample without salt (magenta circles) and 100 μM sample with 50 mM NaCl (blue circles). Solid and dashed dark blue lines denote the net charge and charge of the polyhistidine tag, calculated based on the measured pKa magnitudes. **D—**measured pKa of Glu, Asp sidechains and C-terminal residue of RIP2CARD. Red bars denote the sidechains which are engaged in salt bridges.

This study reveals a rather peculiar pH-dependent behavior of the domain. First of all, at pH below 3.7 the protein is partially unfolded—a set of new narrow and intense signals appear in the random-coil region of the spectra (S2 Fig). At pH above 4.3 the quality of the spectra starts to decrease dramatically and signals vanish, which is accompanied by the changes in the solution physical properties—it becomes very stiff and gel-like. At pH above 5.0 only 40–50 μM of the monomeric soluble protein is retained. At pH above 6.0 the protein precipitates, and only 10–20 μM of the RIP2CARD is preserved in solution (Fig 3C). This precipitation is reversible, the protein can be redissolved at pH 3.5, providing the same quality of NMR spectra and signal pattern in HSQC, which indicates the proper folding of the CARD domain. Moreover, the quality of spectra is depending on the ionic strength of the solution and the concentration of the protein. While in the presence of 50mM NaCl only 20–40 μM of the protein is retained in solution at pH 5.0–6.0, in the salt-free and dilute samples as much as 100 μM of the protein can be observed at pH 5.5. The concentrated salt-free sample also performs much worse than the dilute one at pH above 5.0. To understand the reason of such behavior, we measured pKa of all Glu and Asp sidechains, most of which appeared to lie in the range 3.6–4.0, and calculated the charge profile of the protein (Fig 3C and 3D). At pH 4.2 70% of anionic sidechains are already charged, and above 5.0 the overall charge of the protein is determined exclusively by the state of the histidines in the polyhistidine tag. The structure of RIP2CARD is not changed at neutral pH, according to the NMR spectra (S3 Fig). Thus, the precipitation of RIP2CARD is caused by the change of the net charge of the protein. At low pH, the net charge is +21 and electrostatic repulsion prevents the protein oligomerization. However, at pH above 4.2 the net charge is reduced to +10 and further to +7 at pH above 5.0. This is not enough to repel the proteins, and they start to form multiple contacts and high-order oligomers, which is expressed in the gel-like behavior of the solution. When the His-tag becomes neutral and the charge is further reduced, the protein precipitates since the very high order oligomers are formed. This hypothesis is in the agreement with the expressed effects of the ionic strength on the precipitation. Thus, we conclude that the ability to form high-order oligomers is an intrinsic property of rat RIP2 CARD domain.

The pH titration also allowed detecting the salt bridges that stabilize the fold of the domain. Out of all positively charged residues, only R443, R487, R502 and K485 sidechains are sensitive to the pH changes and these residues are likely to be engaged in ionic interactions (Fig 3A). According to the structure, R443 is close to the D491 (3–6 A between the terminal heteroatoms), К487—to E644 (4–7 A), R502—to both E498 (3–7 A) and E499 (4–7 A) and K485—to E452 (3-6A) (Fig 3B). Thus, five ionic contacts were found. As for the relevance of the obtained structure—almost all sidechains are already charged at pH 4.2, and the overall appearance of HSQC spectra does not change upon titration to pH 6 (S3 Fig). Since the core CARD domain does not contain any histidines, we conclude that the ionogenic state of the protein at pH 4.2 is close to physiological and the structure is thus native.

## Discussion

### Comparison of rat and human RIP2 CARD structures

**Fig 4:**
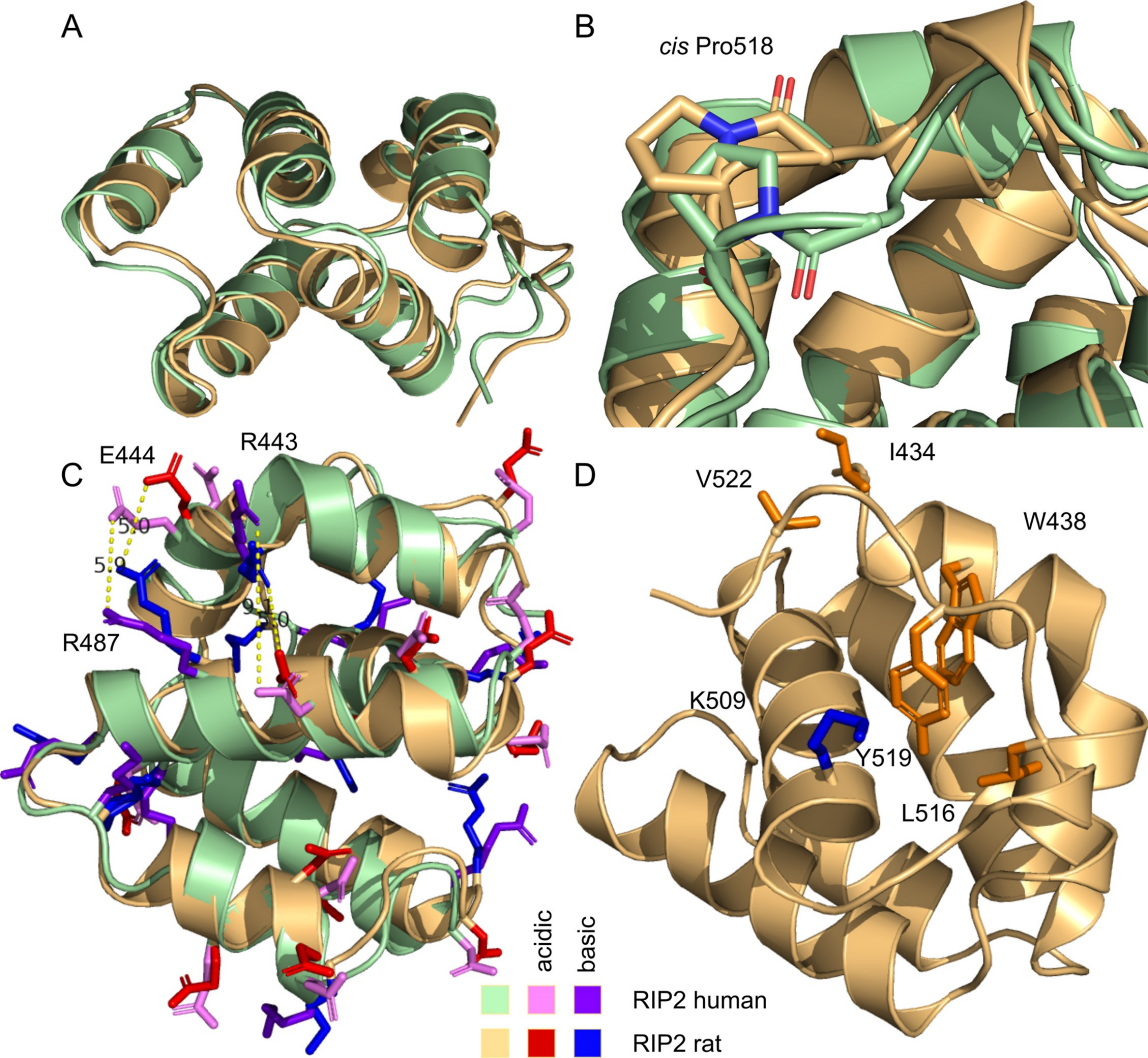
Comparison of human (pale green, pink, magenta) and rat (yellow, red, blue) RIP2 CARD domain structures, reported here and in the work by Lin et al [13]. **A,B,C**—overlay of the structures in ribbon representation. **B** demonstrates the different conformation of the P518 peptide bond. **C** shows the differences in the positions of charged residues in H1. **D—**structure of RIP2CARD with indicated and assigned residues that are important for the proper packing of C-terminal residues.

As soon as the structure of RIP2CARD is determined, it would be interesting to compare it to the other known structures of CARDs in order to find the determinants of both the fold and activity of the protein. First, we compared the obtained structure to the recently published conformation of the human RIP2 CARD (PDB ID 2N7Z, [13]) (Fig 4A). Human and rat proteins are highly homologous with only four substitutions, three of which are synonymous. Two structures appeared to be almost identical, with backbone RMSD equal to 1.3 A. This is a low value, but it exceeds the RMSD within the set of the calculated structures. On the other hand, we found one major difference: while in our structure Q517-P518 peptide bond was in *cis* configuration, which is strongly supported by the experimental data, the same is not observed in the human CARD. This difference results in the significant deviation of the C-terminal region conformation between the two structures (Fig 4B). We claim that the difference is a result of the error in the 2N7Z structure. *Cis* or *trans* configuration of X-Pro peptide bond can be easily established based on the difference between the chemical shifts of Cβ and Cγ nuclei of the Pro residue [33]. We observe the difference of 10.1 ppm (4.1–4.7 ppm for other 4 *trans* Pro residues of RIP2CARD), which corresponds to the 100% probability of *cis* configuration. Similarly, chemical shifts, deposited with the PDB entry 2N7Z, report the Cβ-Cγ difference of P86 (homologous to P518 in RIP2CARD) as large as 10 ppm, which definitely corresponds to the *cis* state. Thus, Q85-P86 bond in 2N7Z should also be in the *cis* state, but it is not. In other words, the 2N7Z structure is erroneous in the region of the C-terminal tail, because the authors failed to recognize the *cis* configuration of the Q85-P86 peptide bond and it was considered in the default *trans* state.

Additionally, some minor differences are observed in the region of the H1 kink. In 2N7Z, the residue corresponding to R443 in RIP2CARD is directed slightly outwards. It disrupts the salt bridge with D491, which is not formed in the specified structure, while its presence is confirmed by our pH titration analysis (Fig 4C). These minor deviations are, most likely, the result of the different approach to the structure calculation used in the current work and in the work by Lin at al. The 2N7Z model is based exclusively on the NOE data, while we rely on 123 extra J-couplings, which provide the more precise local conformation of both backbone and sidechains. Thus, the 2N7Z structure contains an essential error and is less determined by the experimental data, therefore, the present structure needs to be used for homology modeling, docking, and analysis of the protein-protein interactions.

### Homologous CARD domains

**Fig 5:**
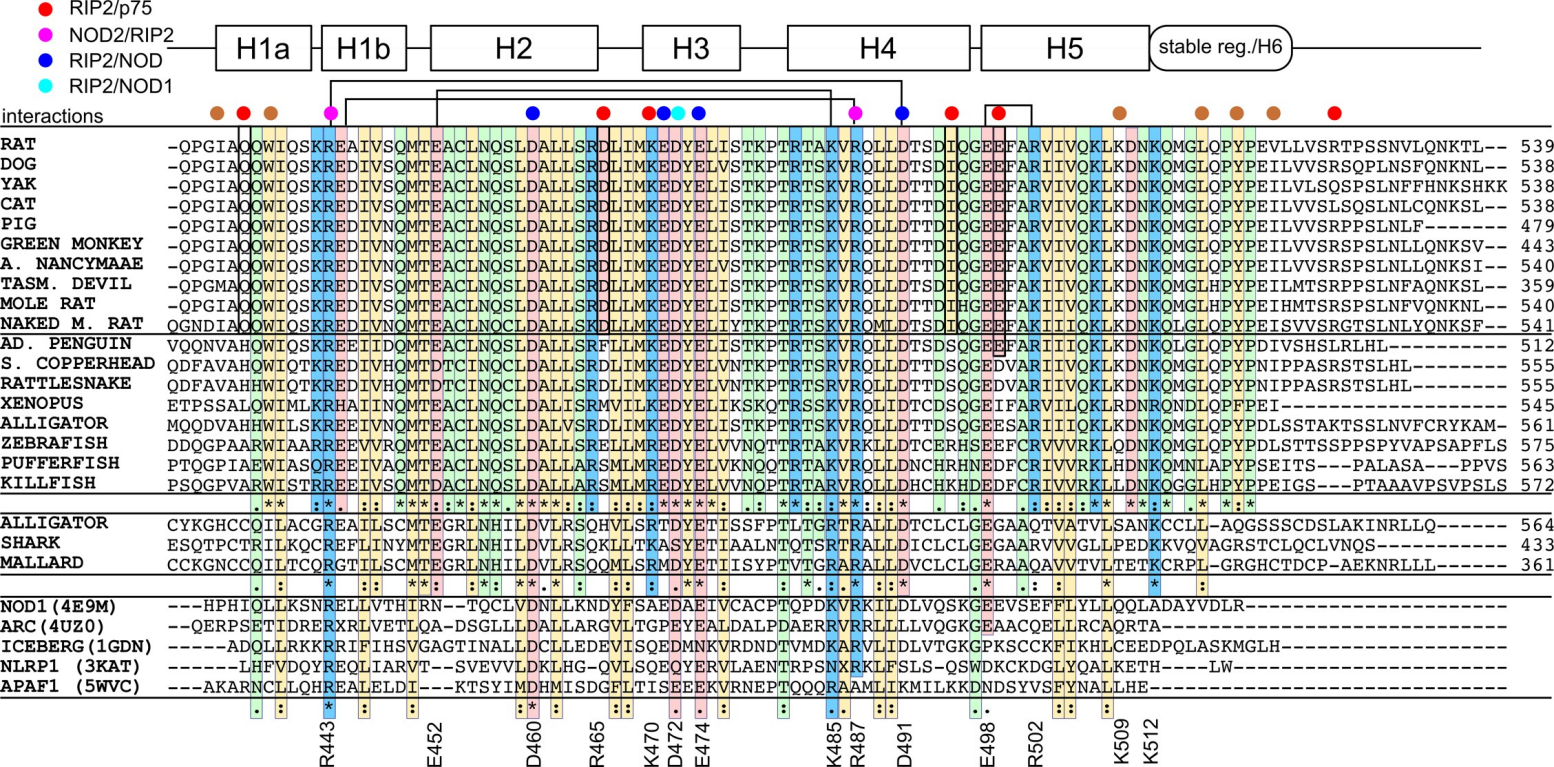
Homologs of RIP2CARD. At top, the aminoacid sequences of homologous to the rat RIP2 RIPK2 proteins are aligned with RIP2CARD sequence. At bottom,CARD domains of the proteins with other functions are aligned based on the 3D structure homology, according to the PDBefold server, PDB IDs are indicated. Conservative residues are highlighted with respect to their physical properties (yellow-hydrophobic, blue—positively charged, red—negatively charged, green—polar or neutral). Lines indicate the salt bridges, observed in RIP2CARD structure. Red, magenta, blue and cyan circles denote the residues which were shown important for the human RIP2-p75 [13], NOD2/RIP2, RIP2/NOD1/NOD2 and exclusively RIP2/NOD1 interactions [15]. Brown circles denote the residues, important for the unique packing of the RIP2CARD C-terminal residues.

Apart from the human RIP2 CARD, we have found several highly similar structures of other CARDs, including NOD1 CARD (PDB ID: 4E9M, RMSD: 1.4A), ARC (activity-related cytoskeleton protein, 4UZ0, 1.8A), IL-1β generation inhibitor Iceberg (1GDN, 2.1 A), NLRP1 (3KAT, 1.9 A) and APAF1 (5WVC, 2.1 A). Despite the different functions, these proteins revealed the surprising identity of their CARD conformation (position and length of helices H1-H5). In order to find the sequence motifs that are responsible for the CARD fold, we performed a simple bioinformatic analysis. We aligned the sequences of several CARDs of RIP2 proteins from various species and aligned the listed CARDs with different function but similar fold, based on the 3D structures (Fig 5). It appeared that most residues that are conserved in this dataset are hydrophobic and form the core of the CARD domains. However, two charged residues are found in all CARD domains—arginine, corresponding to R443, and aspartate, corresponding to D460. We initially hypothesized that R443 is necessary to stabilize the kink in the helix H1 due to the salt bridge with D491, however, no such interaction is observed in ARC1, NLRP1 and APAF1, while the H1 kink is preserved. Apparently, these residues are functionally important. The main function of CARDs is to take part in the CARD-CARD or CARD-DD interactions. These interactions are known to have the ionic nature: surfaces of most CARDs reveal the expressed asymmetry of electrostatic properties—extended positively and negatively charged regions are observed (b-face and a-face) [34]. In the case of RIP2CARD the b-face is formed by R443 and R487—residue which is also conserved in most CARD domains. And a-face is provided by D460 and the cluster of anionic sidechains E471-E474, these residues were shown to be important for RIP2 CARD interactions with the NOD1 and NOD2 CARD domains [15]. Thus, the general fold of the CARD domain is determined by the distribution of polar/hydrophobic properties of aminoacid residues, but not by the charged residues, which are responsible for the function of the domain.

Looking at the RIP2 CARD domain sequences, we found them to be highly conserved. The RIP2 CARD has a unique fold—instead of the sixth helix, like in most other CARD domains, it contains an unstructured but stable C-terminal region, which is tightly packed against the helices H1 and H5. The conformation of the tail is supported by the stacking between the aromatic rings of W438 and Y519, π-cation interactions of the latter with K509 and hydrophobic contacts of L516, I434 and V522. As one can see W438, L516 and 518-P[Y,F]P fragment are highly conserved in most RIP2 CARD domains, suggesting that the organization of the C-terminal region is functionally important and preserved in all species, expressing the RIP2 protein. However, there are several proteins found in amphibia, reptilia and fishes, which are annotated as RIP2, but reveal low homology to the mammalian protein and lack these important residues. Moreover, the genome of these species usually contains another protein, annotated as RIP2 and demonstrating all characteristic features of the mammalian CARD domain. This allows us to conclude that these outlying RIP2 kinases are homologous to RIP2 proteins, but have a different structure of CARD domain and apparently the different function/interaction partners. The motif, containing W at the N-terminus and L-X-P-[Y,F]-P at the C-terminus is thus characteristic for the CARD domains of RIP2 kinases, responsible for their unique structure. As for the charged residues, one can observe that all residues, responsible for the interaction with NOD1 and NOD2 are conserved in all RIP2 CARD domains, while the residues that were shown necessary for the p75 binding [13] are conserved mainly in the mammalian proteins.

### Oligomerization of CARD domains

As we have already stated, RIP2 CARD domain has an intrinsic propensity to homo-oligomerize at neutral pH. Our data allow the numerical estimation of the dimerization constant. Assuming the monomer-oligomer equilibrium, in the excess of the oligomeric protein, the dimerization constant should not exceed the concentration of monomer in solution, which is below 10 μM at neutral pH. Apparently, this property is also relevant for the human RIP2. It is not directly stated in the work by Lin et al [13], but the structure of human RIP2 CARD domain was investigated in the absence of buffer and unspecified pH, unlike the other proteins in the work which were studied at explicitly stated neutral pH and in 30–50 mM salty buffers. Additionally, another work [15] reports the inability to produce the soluble sample of human RIP2 CARD domain and the incorrect structure of the protein after the refolding procedure, however, the refolding protocol and used pH values are not provided. This oligomerization propensity may be restricted by the presence of kinase and intermediate domains since the full-size RIP2 protein is known to form homodimers [11].

**Fig 6:**
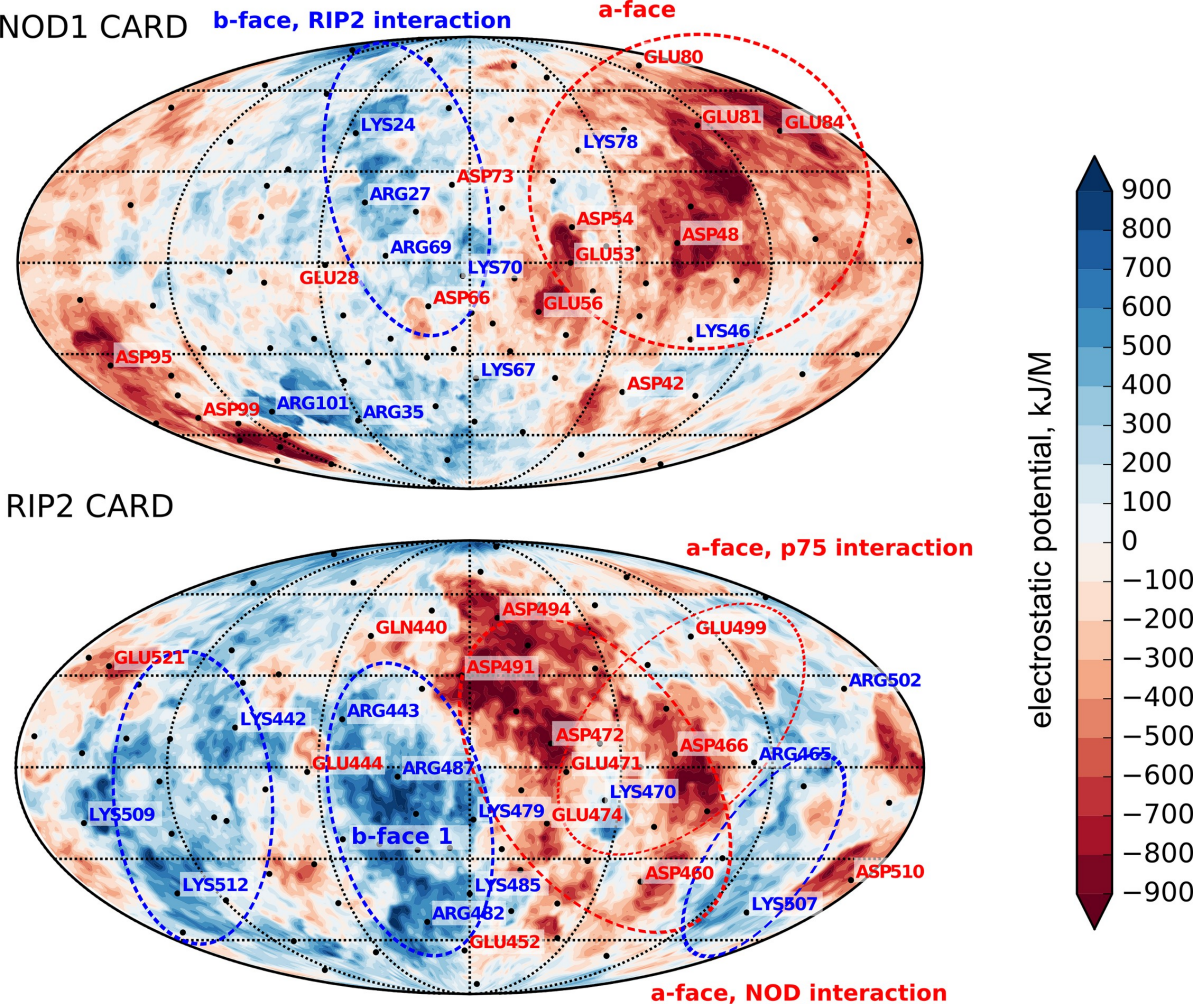
Electrostatic parameters of RIP2CARD and NOD1 CARD. The maps of electrostatic potential distribution on the Van-der-Waals surface of NOD1 CARD (on top) and RIP2 CARD domains are shown. Two molecules were aligned based on their structures. Red regions correspond to the negative charge, while blue regions—to the positive one. Areas of proteins surfaces involved or possibly involved in the various kinds of protein-protein interactions are indicated by dashed ellipses.

Several reasons could be suggested for the RIP2CARD oligomerization: disulfide cross-linking, electrostatic and hydrophobic interactions. Disulfide cross-linking could be ruled out: C454 is in the reduced state, according to the Cβ NMR chemical shift (25 ppm) [35], and the precipitation is reversible, which is impossible in the case of covalent bond formation. Hydrophobic interactions do not agree with the properties of the RIP2CARD surface—it is highly polar. Thus, electrostatic interactions are likely to cause the RIP2 oligomerization, which agrees well with the pronounced effect of salt concentration on the RIP2CARD solubility. The analysis of RIP2CARD surface charge may help to understand the tendency of the domain to oligomerize. We employed the Protein Surface Topography approach [27,28] to compare the electrostatic potential distribution on the surface of NOD1 and RIP2 CARD domains (Fig 6). The maps show that two domains are substantially different. NOD1 CARD provides the expressed negatively charged (a-face) surface and a less expressed positively charged site (b-face), with the latter taking part in the NOD1/RIP2 interaction. In contrast, the surface of RIP2CARD is rather patchy. At least three highly positively charged faces of RIP2CARD may be observed, together with the prominent a-face, which includes two intersecting interfaces for the p75 death domain and NOD1/NOD2 CARDs. Apart from the extended a-face, several negatively charged patches may be found for the protein under investigation. It is possible, that such electrostatic parameters permit RIP2CARD to form multiple protein-protein contacts in various orientations. This is reflected in the extremely high viscosity of the RIP2CARD solutions at pH above 5.5.

## Conclusion

In the present work, we report the high yield expression, the refolding protocol and resolve the structure of rat RIP2 CARD domain, involved in a variety of signaling events in the cell. The obtained structure reveals several important differences to the previously published conformation of the homologous human protein. Using solution NMR, we characterized the intramolecular mobility and pH-dependent behavior of RIP2 CARD and found the propensity of the protein to form high-order oligomers at physiological pH while being monomeric in the acidic environment. The oligomerization of protein may be explained, based on the electrostatic properties of its surface. Analysis of the obtained structure and sequences of homologous proteins reveals the residues which are significant for the unusual fold of RIP2 CARD domains from different species. The development of high-throughput refolding protocol together with the production system based on the E. coli cells and found reasons for the protein oligomerization provides an opportunity to design the stabilized variants of RIP2 CARD domains, which could be used to study the structural details of RIP2/NOD1/NOD2 interaction and rational drug design.

## Supporting information

S1 Fig Protein expression of RIP2CARD fusion constructs. Whole cell lysates and soluble protein fractions (after centrifugation at 14000 g) were analyzed using 12% SDS-PAGE gels. Cells were grown at 28°C until the culture reached an OD600 of ~0.6 and proteins expression were induced by IPTG (0, 0.1 and 1 mM), then cultivation continued overnight at 37°C and 13°C. To estimate the protein solubility the cell pellet was resuspended in buffer (20 mM Tris-HCl, pH 8.0, 100 мМ NaCl, 100 μM PMSF, 1 mM EDTA, 0.5% Triton X-100), lysed by ultrasonication on ice and centrifuged at 14000 g for 20 minutes. The 20 μl of culture were analyzed in each lane. The proteins of interest were indicates by arrows. A-D: analysis of Thioredoxin, SUMO, GST and His-tag constructs of RIP2CARD, respectively.
(TIF)

S2 Fig ^1^H,^15^N-HSQC spectra of 300 μM RIP2CARD acquired at 30°C, pH 3.3, 4.0 and 5.1. Spectra were recorded identically and are plotted with the equal contour parameters. A portion of peaks, which appear due to the partial pH-induced unfolding of RIP2CARD are indicated on the left panel.
(TIF)

S3 Fig RIP2CARD structure is the same at pH 4.2 and 6.0. Fragments of ^1^H,^15^N-HSQC (A) and ^1^H,^13^C-HSQC (B) spectra of RIP2CARD, recorded at pH 4.2 (red) and 6.0 (blue). While position of some amide cross-peaks change due to the deprotonation of Asp and Glu sidechains, the pattern of methyl group cross-peaks is retained, which confirms the identity of the domain structure at moderately acidic and neutral pH.
(TIF)

S1 Table Input data and statistics for the 20 best NMR structures of RIP2CARD. (DOCX)
