# Supplementary figures and images for: CARD domain of rat RIP2 kinase: Refolding, solution structure, pH-dependent behavior and protein-protein interactions
Source: PLoS One. 2018 Oct 23;13(10):e0206244. doi: 10.1371/journal.pone.0206244 (PMC6198988; doi:10.1371/journal.pone.0206244)

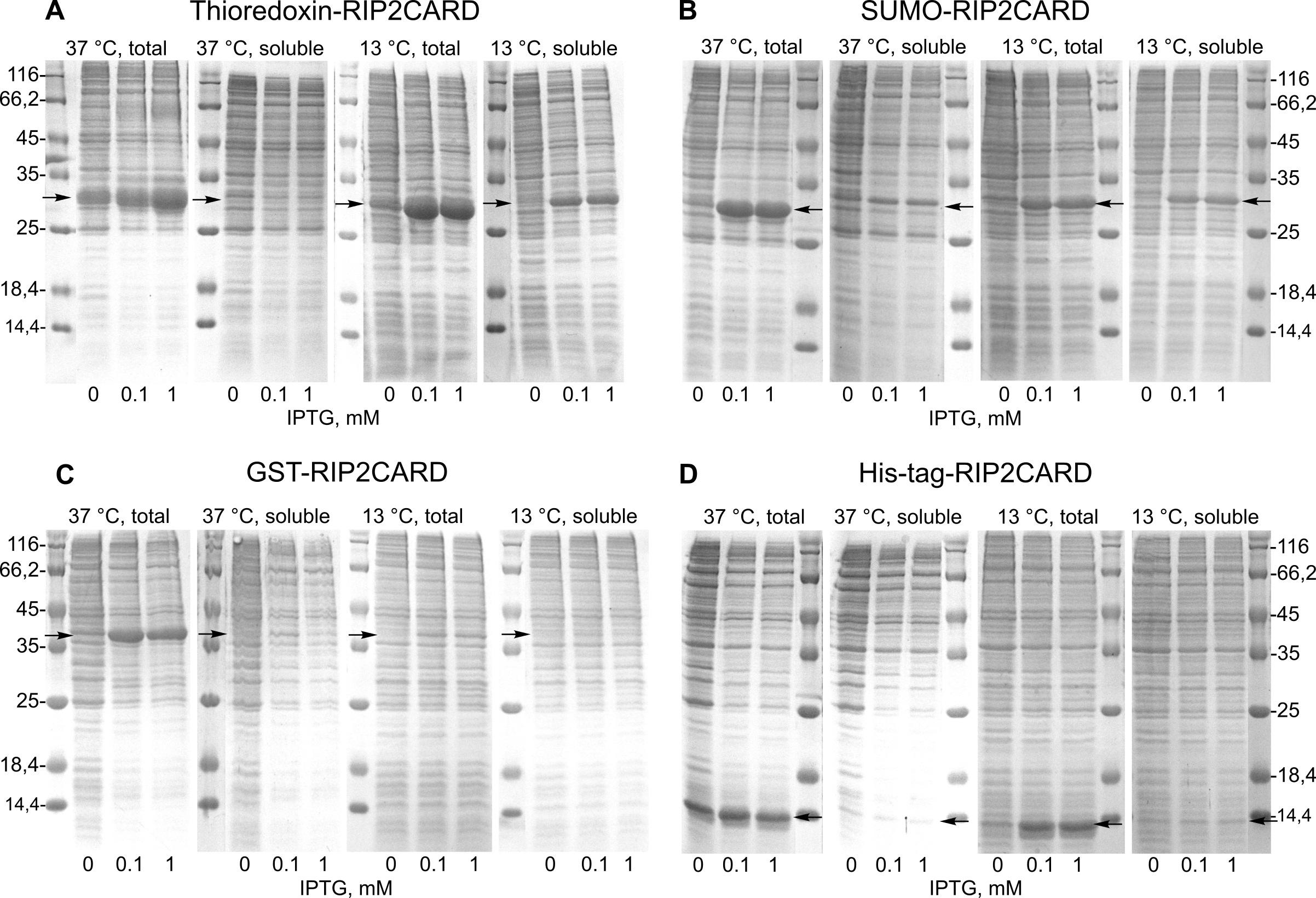

Supplement: S1 Fig — Whole cell lysates and soluble protein fractions (after centrifugation at 14000 g) were analyzed using 12% SDS-PAGE gels. Cells were grown at 28°C until the culture reached an OD600 of ~0.6 and proteins expression were induced by IPTG (0, 0.1 and 1 mM), then cultivation continued overnight at 37°C and 13°C. To estimate the protein solubility the cell pellet was resuspended in buffer (20 mM Tris-HCl, pH 8.0, 100 мМ NaCl, 100 μM PMSF, 1 mM EDTA, 0.5% Triton X-100), lysed by ultrasonication on ice and centrifuged at 14000 g for 20 minutes. The 20 μl of culture were analyzed in each lane. The proteins of interest were indicates by arrows. A-D: analysis of Thioredoxin, SUMO, GST and His-tag constructs of RIP2CARD, respectively. (TIF) [file pone.0206244.s001.tif]

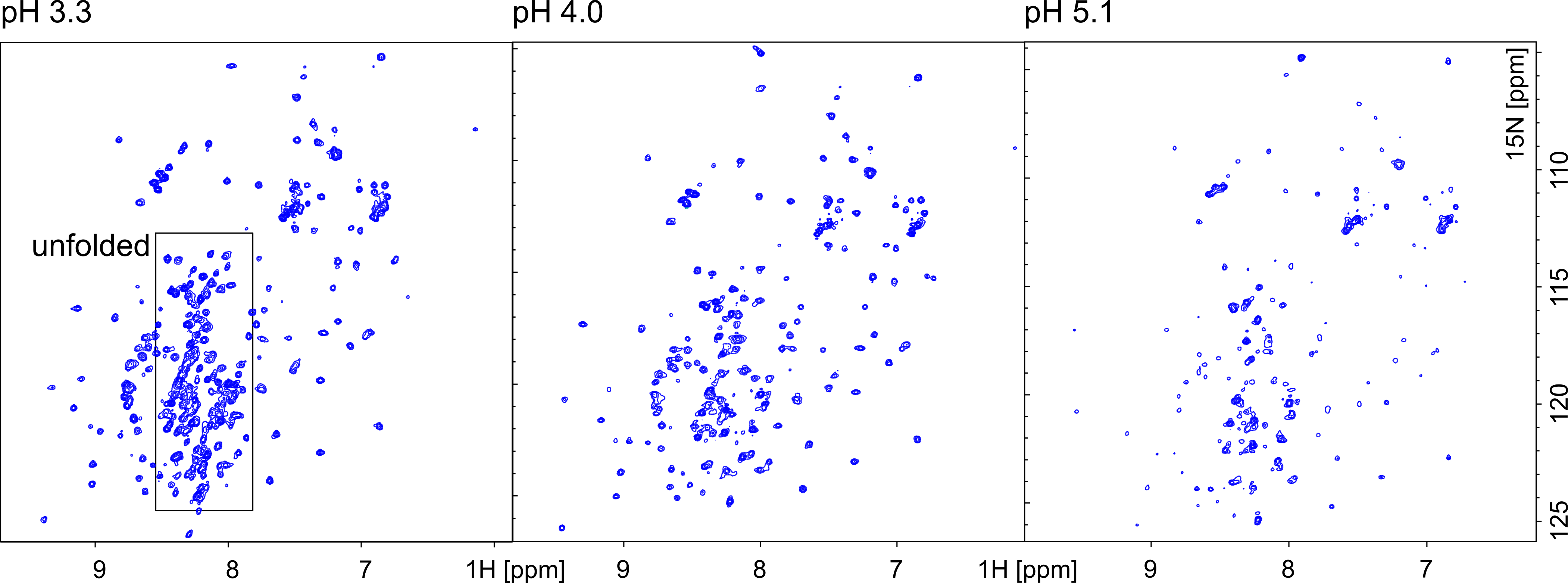

Supplement: S2 Fig — Spectra were recorded identically and are plotted with the equal contour parameters. A portion of peaks, which appear due to the partial pH-induced unfolding of RIP2CARD are indicated on the left panel. (TIF) [file pone.0206244.s002.tif]

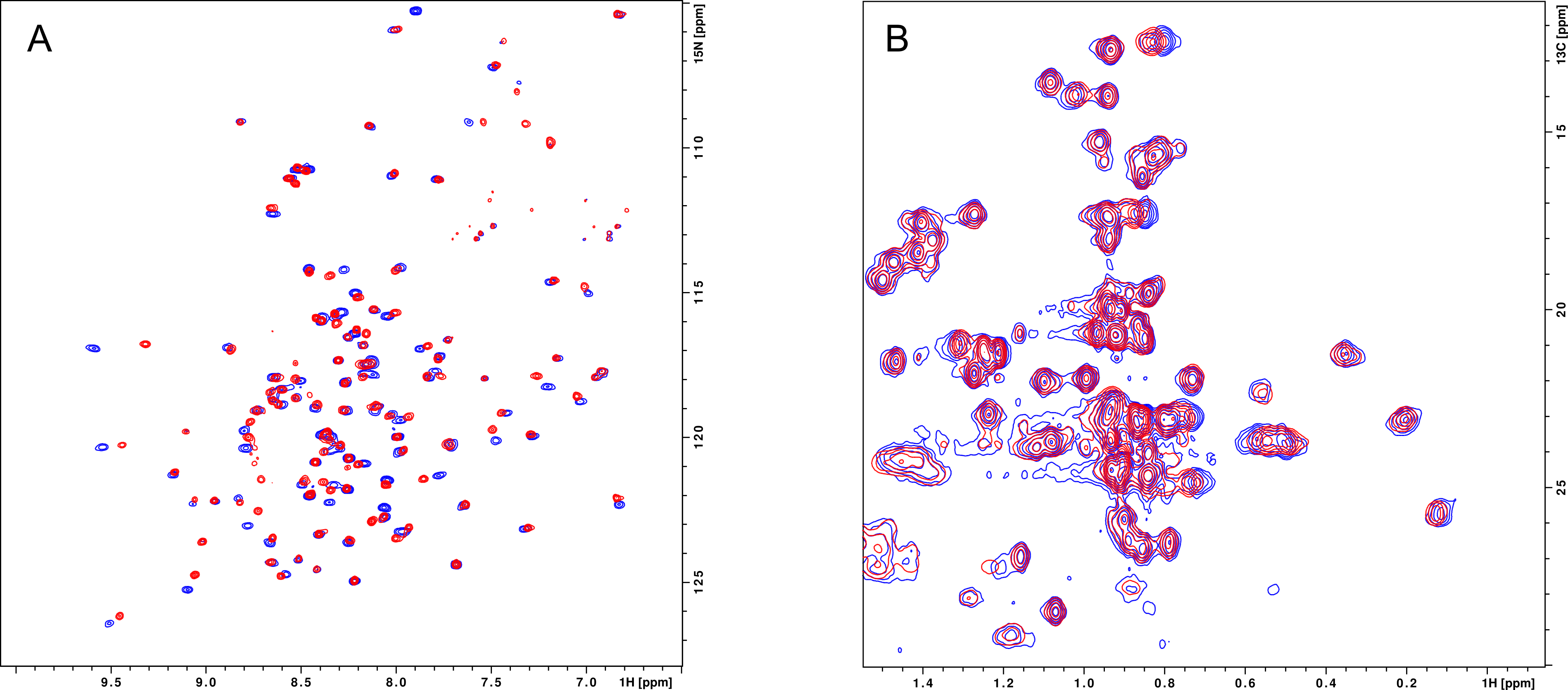

Supplement: S3 Fig — Fragments of 1H,15N-HSQC (A) and 1H,13C-HSQC (B) spectra of RIP2CARD, recorded at pH 4.2 (red) and 6.0 (blue). While position of some amide cross-peaks change due to the deprotonation of Asp and Glu sidechains, the pattern of methyl group cross-peaks is retained, which confirms the identity of the domain structure at moderately acidic and neutral pH. (TIF) [file pone.0206244.s003.tif]
