# Supplementary material for: CARD domain of rat RIP2 kinase: Refolding, solution structure, pH-dependent behavior and protein-protein interactions
Source: PLoS One. 2018 Oct 23;13(10):e0206244. doi: 10.1371/journal.pone.0206244 (PMC6198988; doi:10.1371/journal.pone.0206244)
Supplement: S1 Table — (DOCX) [file pone.0206244.s004.docx]

**S1 Table.** Input data and statistics for the 20 best NMR structures of RIP2CARD.

| **Distance and Angle restraints** | |
| --- | --- |
| Total NOE contacts | 1678 |
| intraresidual | 610 |
| sequential (\|i-j\|=1) | 381 |
| medium-range (1<\|i-j\|<4) | 386 |
| long-range (\|i-j\|>4) | 301 |
| J-couplings | 123 |
| ^3^J_HNHα_ | 75 |
| ^3^J_CγC'_ | 24 |
| ^3^J_CγN_ | 24 |
| **Total restraints/per residue (433-524):** | **1801/19.8** |
| **Statistics for calculated structures** | |
| Structures calculated/selected | 100/20 |
| CYANA target function (Å^2^) | 2.06 ± 0.24 |
| **Violations of restraints** | |
| Distance (>0.2 Å) | 3 |
| Dihedral angles (>5 °) | 0 |
| **RMSD (Å) regular structure (434-517)** | |
| Backbone | 0.49±0.07 |
| All heavy atoms | 1.14±0.08 |
| **RMSD (Å) full domain (433-524)** | |
| Backbone | 0.73±0.18 |
| All heavy atoms | 1.36±0.17 |
| **Ramachandran analysis** | |
| Residues in favored regions (%) | 80.2 |
| Residues in allowed regions (%) | 100 |
